# Functioning of the children with hydrocephalus

**DOI:** 10.1007/s13760-020-01280-y

**Published:** 2020-01-23

**Authors:** Lucyna Szefczyk-Polowczyk, Marek Mandera

**Affiliations:** Department of Pediatric Neurosurgery, Medical University of Silesia, Katowice, Poland

**Keywords:** Hydrocephalus, Children, Barthel scale, Functioning, Outcome

## Abstract

The objective of the study was to examine selected aspects of the functioning of children treated for hydrocephalus, with particular emphasis on their degree of independence. Analysis of the medical records of patients treated for hydrocephalus in the Department of Pediatric Neurosurgery in Katowice in 2000–2010. The Barthel scale was used to assess the children’s independence. Information on the children’s functioning was obtained directly from their parents using a questionnaire developed by the author. The parent group comprised 131 people, including 110 women (85%) and 21 men (15%). The study group comprised 131 children treated for hydrocephalus. In the examined group, 59 children (45%) were fully independent (first degree), 44 (34%) were partially independent (second degree) and 28 (21%) were completely dependent (third degree). Most of the children with congenital and post-inflammatory hydrocephalus attended the generally accessible school (public school), while the majority of the children with posthemorrhagic hydrocephalus attended rehabilitation and educational centers or special schools (*p* < 0.05) The lowest level of independence was found for children with hemorrhagic hydrocephalus who had undergone repeated operations. The child’s degree of independence and the etiology of hydrocephalus determined the type of school the child attended. The study’s results indicate that rehabilitation plays a key role in the lives of children with hydrocephalus.

## Introduction

Hydrocephalus is characterized by its heterogeneous nature, which makes predicting the development of a child with hydrocephalus extremely difficult. The child’s functioning and degree of its independence are related to the individual patient's condition, his/her age, and the etiology of hydrocephalus and coexisting diseases, among other factors. The child’s degree of function may vary from total dependence on other people to full independence.

The neurodevelopmental diagnosis of children who develop hydrocephalus after birth as a consequence of tumors, inflammatory processes or injuries is very difficult and depends on the underlying disease and its neurological complications. These variables make it difficult to predict exactly how the patient’s cognitive development will proceed. Hydrocephalus in children may be accompanied by various disorders affecting the hearing, visual, bone-joint and muscular and the atrial systems, among others [1].

The lack of information regarding the education and interpersonal relations of children treated for hydrocephalus in Poland makes the assessment of their functioning at school age impossible. The absence of such knowledge means that the parents of sick children are not able to adapt mentally to how their child’s future will look and how much independence they can achieve.

### Objective

The objective of this research was to examine selected aspects of the functioning of children treated for hydrocephalus, with particular emphasis on their degree of independence.

## Methods

Analysis of the medical records of patients treated for hydrocephalus in the Departement of Pediatric Neurosurgery in Katowice in 2000–2010 was performed. The Barthel scale was used to assess the children’s independence.

Information on the children’s functioning was obtained directly from their parents using a questionnaire developed by the author. The parent group comprised 131 people, including 110 women (85%) and 21 men (15%). The study group consisted of 131 children treated for hydrocephalus of various etiologies (congenital, posthemorrhagic, post-inflammatory, injury and unclear etiology) and comprised 78 boys and 53 girls aged 3–19 years. Children with hydrocephalus associated with MMC (meningomyelocele) were excluded due to the dominating influence of deficits resulting from MMC on the child’s functioning. For the statistical analysis of the results, the following software was used: PQStat 1.6.0 (PQStat software) and the Excel program of the MS Office package. Results were considered significant when the statistical significance level obtained was *p* < 0.05. Table 1 shows the sociodemographic profile of the study participants.

**Table 1:** Sociodemographic profile of study participants

	Etiology of hydrocephalus in a child											
	Congenital *n* = 58		Post-inflammatory *n* = 21		Post hemorrhagic *n* = 41		Post-traumatic *n* = 3		of unclear etiology *n* = 8		Total [*N* = 131]	
	*n*	%	*n*	%	*n*	%	*n*	%	*n*	%	*n*	%
Age of the child*												
3–6	15	26	9	43	16	39	1	33	0	0	41	31
7–10	17	29	1	5	15	36	2	67	0	0	35	27
11–15	11	19	5	24	4	10	0	0	2	25	22	17
16–18	7	12	2	9	4	10	0	0	2	25	15	11
> 18	8	14	4	19	2	5	0	0	4	50	18	14
Sex of the child												
Male	34	59	12	57	26	63	3	100	3	37	78	60
Female	24	41	9	43	15	37	0	0	5	63	53	40
Total [*N* = 131]	58	44	21	16	41	31	3	3	8	6	131	100

*The age of the child at the time of the survey

The authors declare that they have no conflict of interest.

According to the decision of Ethics Committee of the Medical University of Silesia, (decision KNW/0022/KB/129/13 of 13.06.2013) the project does not require ethics approval as it is based solely on the questionnaire study and is not a medical experiment.

## Results

### Independence of the children according to the Barthel scale

**Fig. 1:**
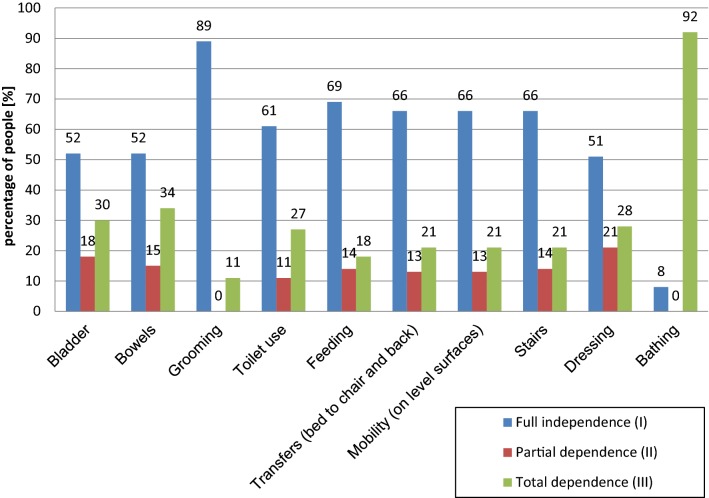
Independence of the children according to the Barthel scale

In the examined group, 59 children (45%) were fully independent (first degree), 44 (34%) were partially independent (second degree) and 28 (21%) were completely dependent (third degree). Figure 1 shows the degree of independence for several categories of activities. Over half of the examined children could independently perform nine out of ten everyday activities.

### The degree of independence and the number of operations

**Fig. 2:**
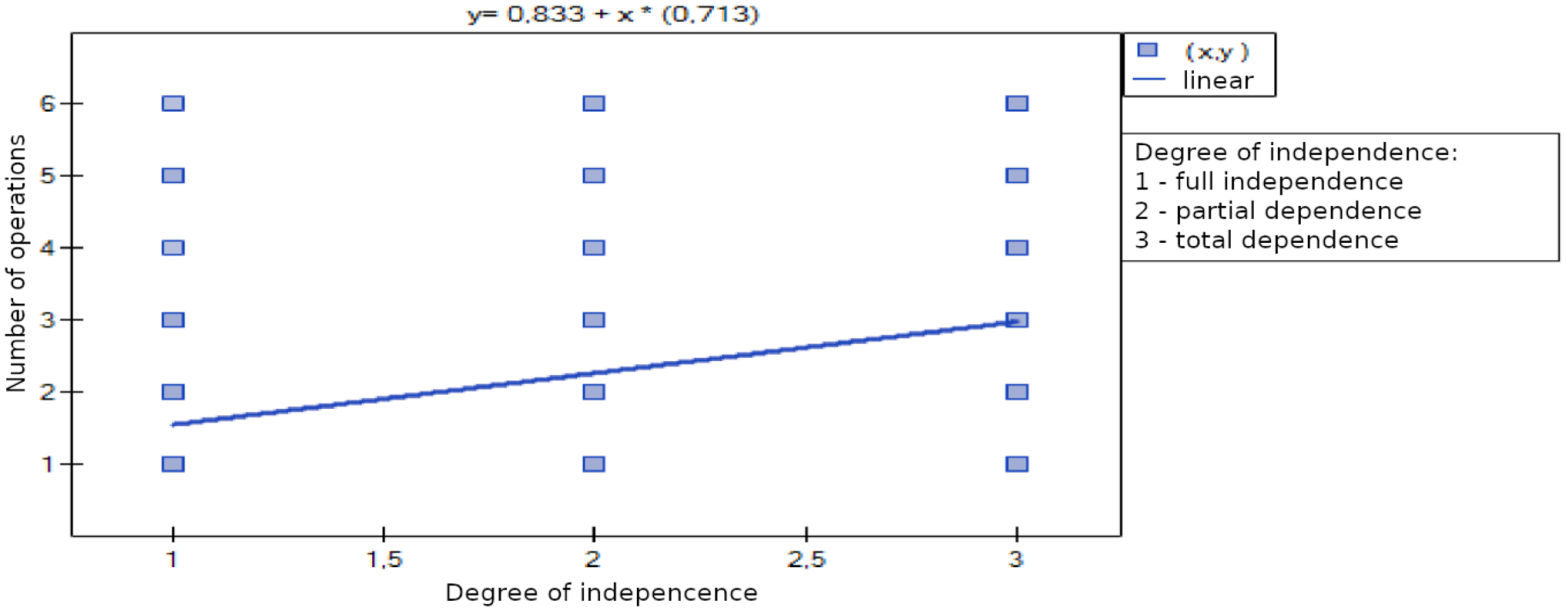
Relationship between the number of completed operations and the degree of independence of the child

A positive trend was observed (*r* = 0.38, confidence interval 0.22 ÷ 0.52, df = 129) between the child’s dependence and the number of operations he or she had undergone (*p* = 0.000005) (Fig. 2).

### Health problems in the children

**Fig. 3:**
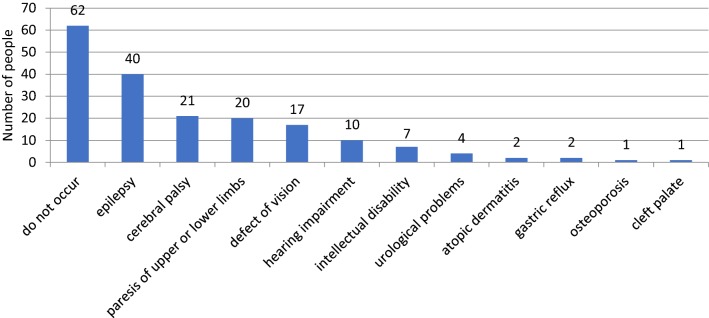
Health problems in the children

The parents were asked if their children currently or previously had other health problems in addition to hydrocephalus. The inquiry was presented as an open question (i.e., the parents could give any answer). The responses of 62 parents indicated that the child had no additional disease burden. Other parental responses were grouped. The answers are presented in Fig. 3.

### Impact of a child’s illness on contact with friends

More than half of the surveyed parents felt that their child’s illness limited contact and meetings with friends (70 people—53%). A slightly smaller percentage of the respondents did not indicate such limitations (61 people—47%). The parents’ subjective opinions regarding the impact of the disease on limiting contacts with friends were analyzed in terms of the child’s degree of independence; the results indicated that children’s lack of independence was related to limitations of contact with friends (Mann–Whitney *U* test, *p* = 0.0001).

### Rehabilitation of children

The child’s degree of independence was associated with his/her rehabilitation. More than half of the examined children were participating in systematic rehabilitation during the survey (80 children—61%), and the remaining 51 children were not (39%).

**Fig. 4:**
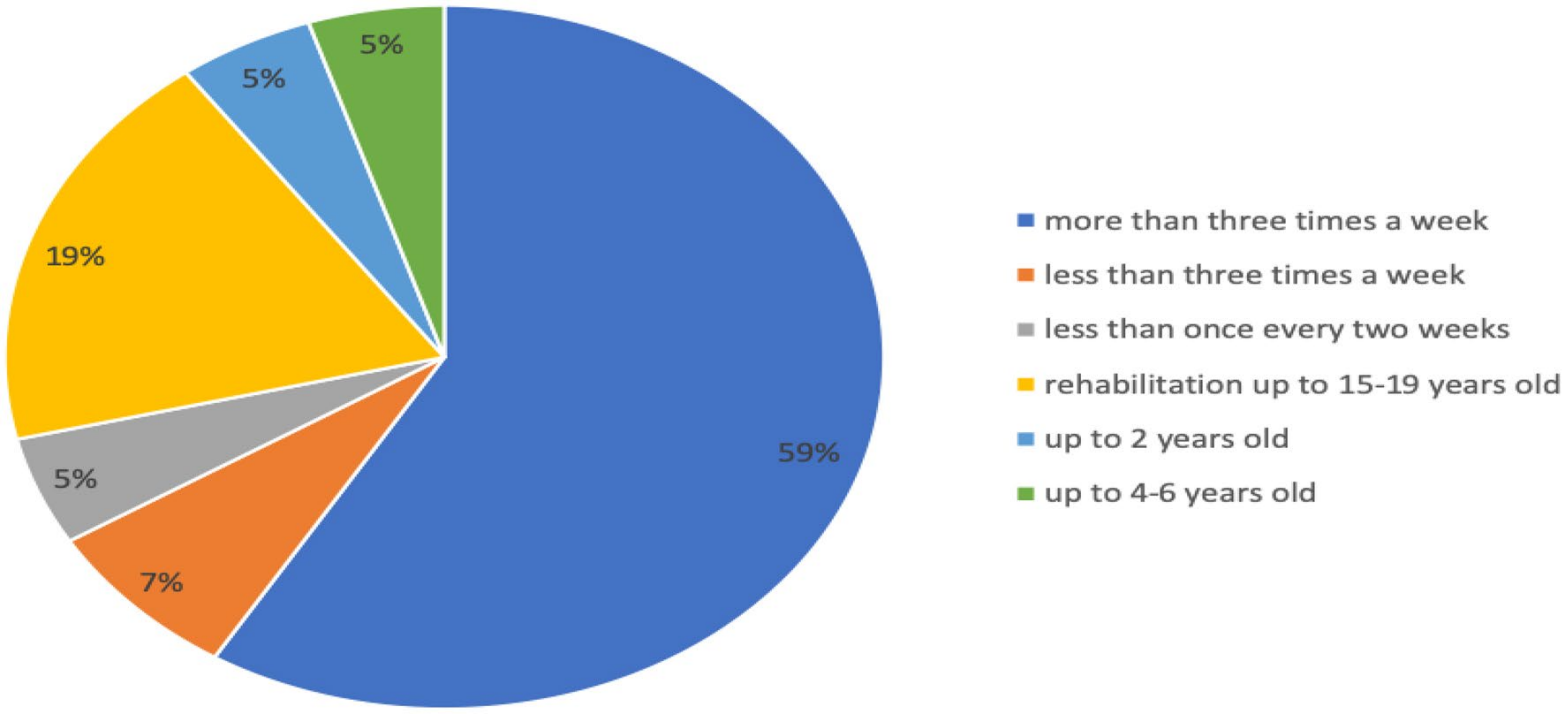
Frequency of rehabilitation of children treated for hydrocephalus

An analysis of the frequency of rehabilitation indicated that the largest group consisted of children attending rehabilitation more than three times a week (47 children—59%). The second largest group included children who had completed rehabilitation but had attended rehabilitation at least twice a week until the age of 15–18 years (15 people—19%). The frequency of rehabilitation for the children treated for hydrocephalus is shown in Fig. 4.

### Treatment from specialist physicians

**Fig. 5:**
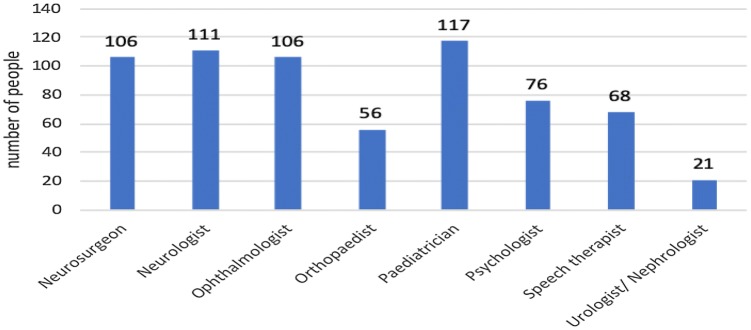
Treatment from specialist physicians

A significant percentage of the children had also been diagnosed with other diseases or dysfunctions that required visits to various specialists (Fig. 5). Most of the children were under the care of a pediatrician and a neurologist. The same number of children (106 children) was treated by a neurosurgeon and an ophthalmologist.

An analysis of frequency of specialist appointments attended by the children with hydrocephalus indicated that appointments were usually made several times a year and most of these visits were for monitoring (57 children—44%). The second most common response was that the children visited specialists every month (35 children—27%); 16% indicated that their children saw a specialist a few times a month, while 12% indicated that specialist visits took place once a year and 2% reported visiting a specialist less than once per year.

### Education of children treated for hydrocephalus

**Fig. 6:**
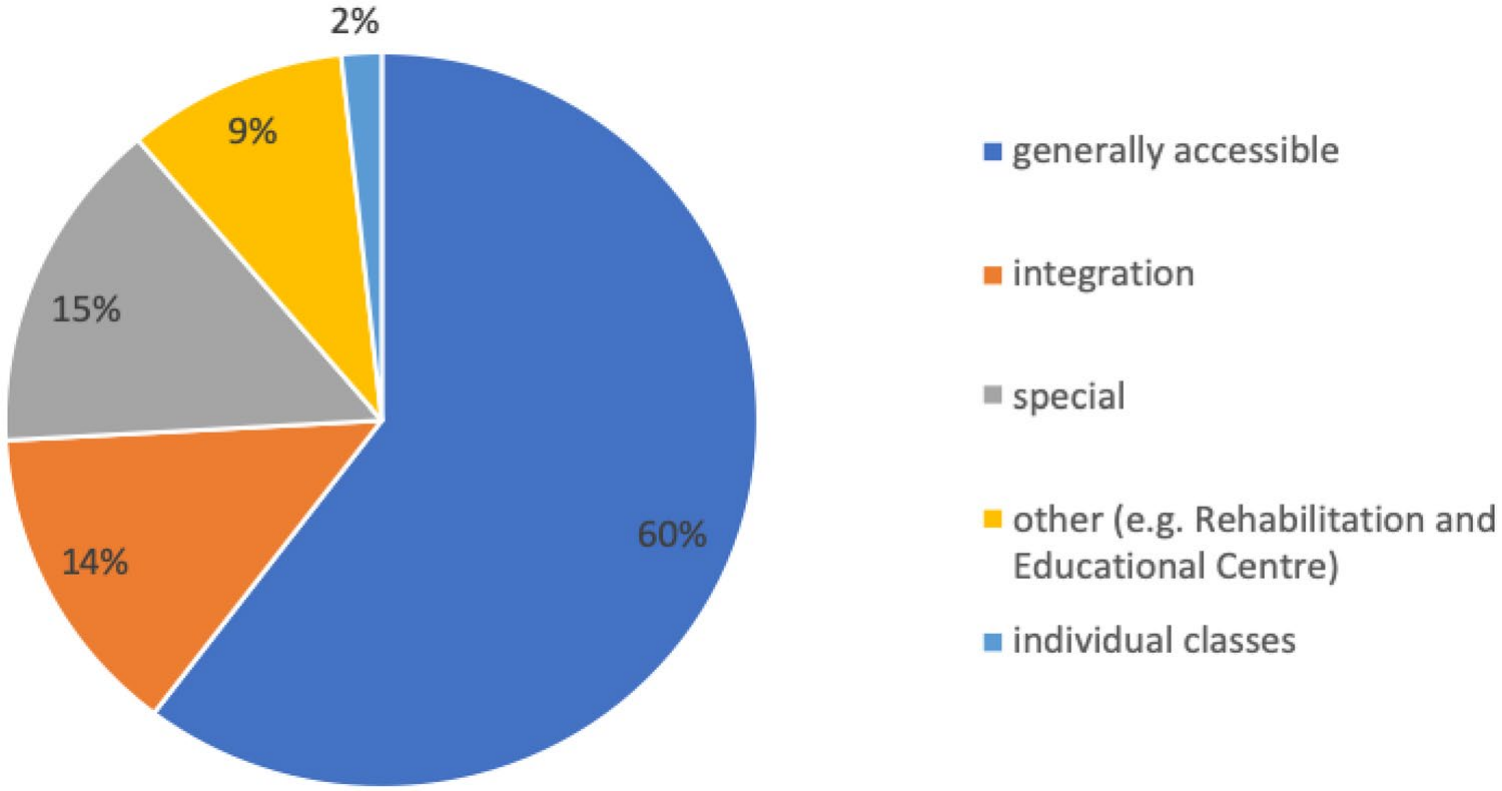
Types of educational facilities attended by the examined children, treated for hydrocephalus

Most of the examined children attended school (78 children—60%). In the case of 13 children, their health condition did not allow them to participate in classes at an educational institution; moreover, these children were not subject to compulsory schooling (10%) due to their age. Thirty percent of the children attended kindergarten. An analysis indicated that most of the studied children attended generally accessible educational institutions (public school). In the case of children with neurological deficits or other developmental limitations, schooling took place at the Rehabilitation and Educational Center (11 children—9%) or in individual classes at home (two children—2%). The types of educational facility that the children with hydrocephalus attended are presented in Fig. 6.

**Fig. 7:**
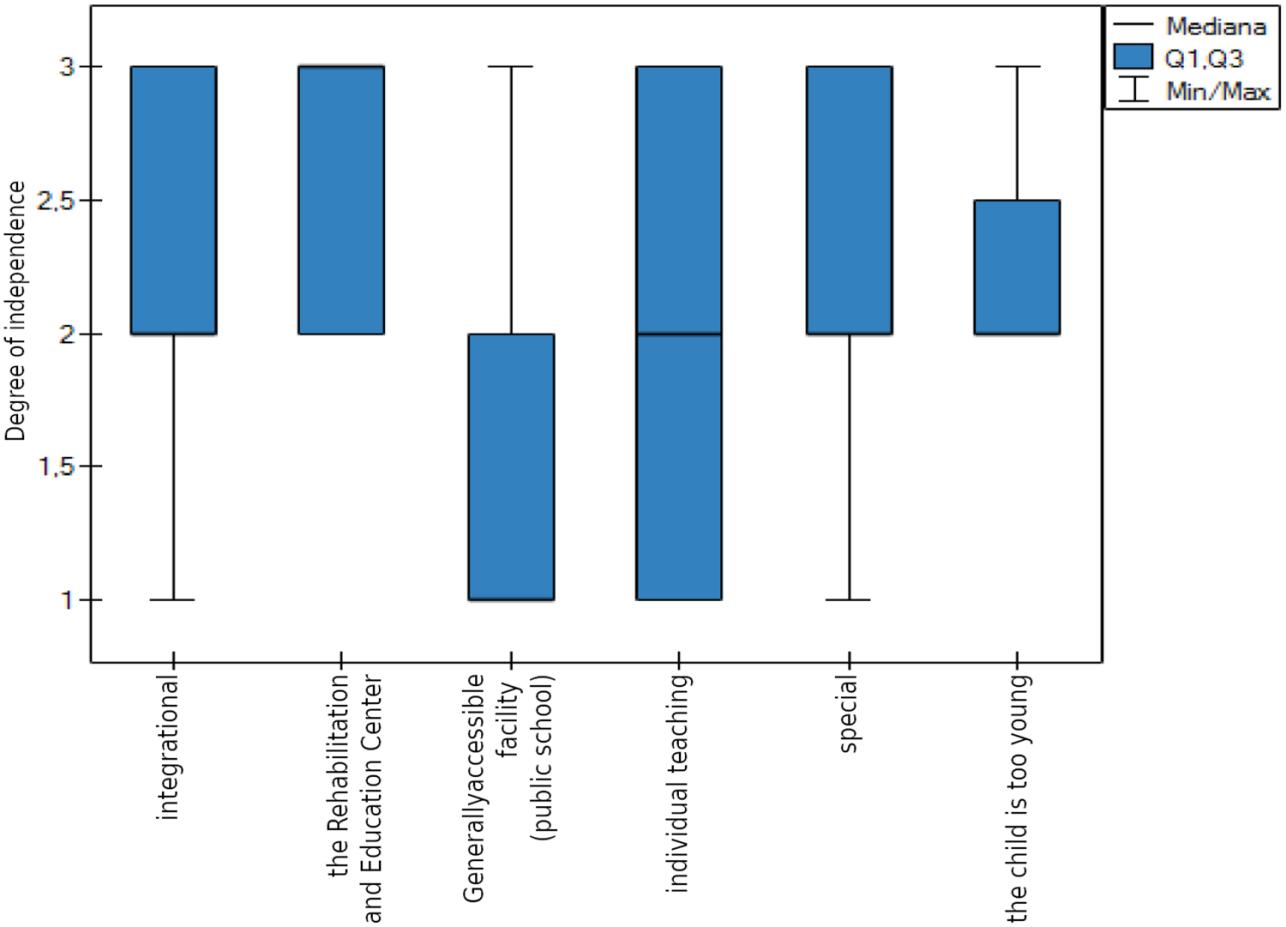
Type of educational facility and the child’s degree of independence

There were statistically significant associations between the child’s degree of independence and the type of educational facility that was attended, *p* < 0.00001 (Kruskal–Wallis ANOVA) Fig. 7.

**Fig. 8:**
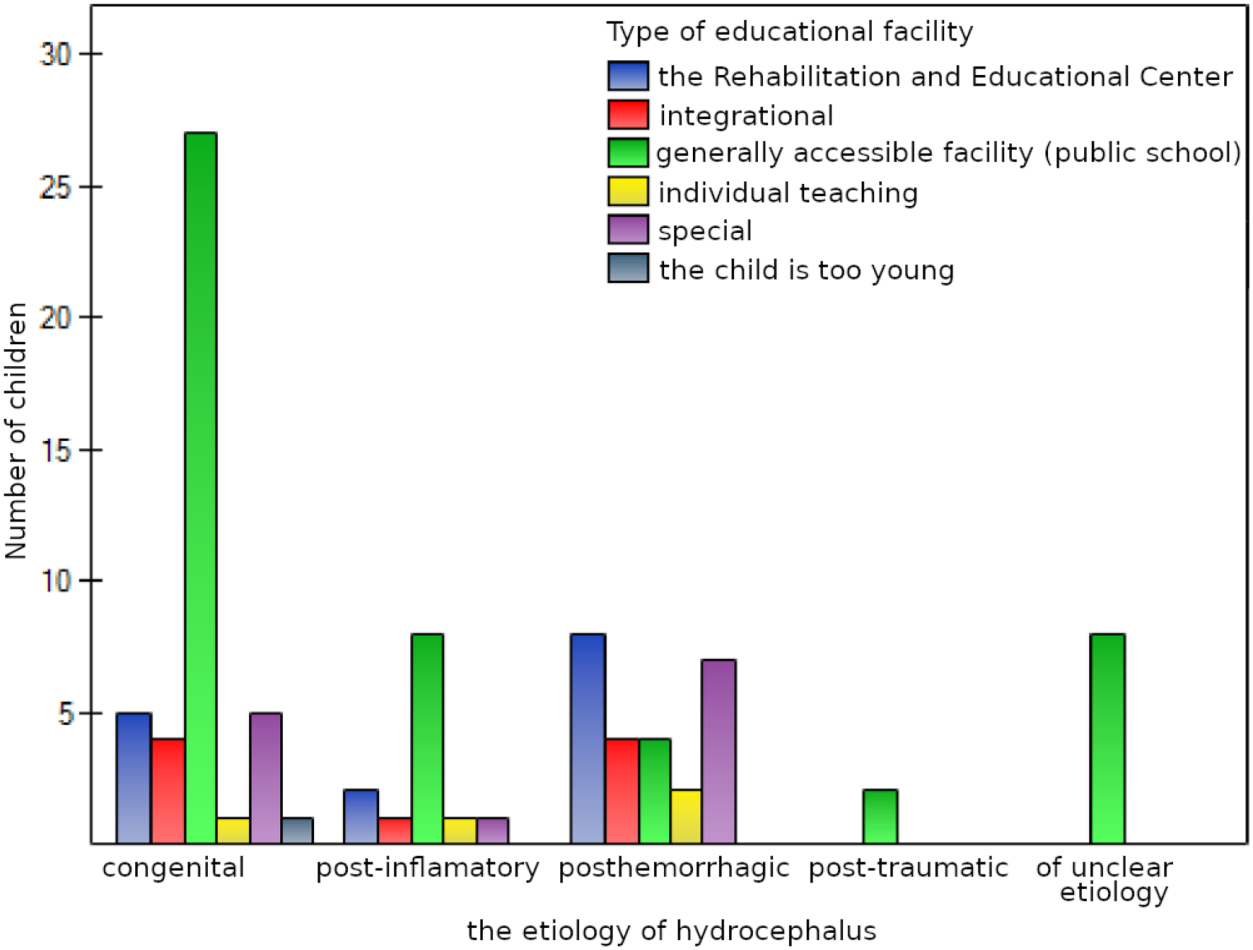
Type of educational facility and the etiology of the child’s hydrocephalus

The majority of the children attended classes based on their age, in the same manner as their healthy peers (70 people—90%). The other children experienced a delayed start of their education (eight people—10%). When associations between the etiology of hydrocephalus and the type of school the children attended were examined, a statistically significant result was obtained. Most of the children with congenital and post-inflammatory hydrocephalus attended a generally accessible school (i.e., a public school), while the majority of the children with posthemorrhagic hydrocephalus attended the Rehabilitation and Educational Center or special schools (*p* < 0.05), Fig. 8.

The most common learning difficulties among the children with hydrocephalus.

The parents of the school-aged children in this study were asked how the child was managing at school and whether there were particularly problematic areas. The inquiry was posed as an open question; the parents had the opportunity to give any answer, and their responses were subsequently grouped. Most of the respondents (35 parents) answered that their child had no learning problems. The next largest group (26 parents) indicated that their child had problems concentrating in school. Graphomotor problems in the children were reported by 23 parents. Other difficulties with school that the children experience included problems with reading (reported by seven parents), science (14 parents) and not participating in physical education (eight parents).

### Contacts with peers

The parents were asked about their children's relationships with their peers. The majority of the children enjoyed meeting with their peers (as reported by 101 parents—77%), while 15% of the parents reported that their children were irritable and shy and 8% reported that their children avoided meeting with other children and that consequently, such contact was very rare. In total, more than half of the surveyed parents admitted that they did not take vacations with their children (79 people—60%). Among those parents, almost 1/3 did not take their children on vacations due to the fear that their child’s health state would deteriorate (39 people—30%). Some of the parents did not take vacations for financial reasons (27%) or for other reasons (3%). The remaining parents indicated that they used to go on vacations with their children (40%).

## Discussion

The degree of independence among studied children with diagnosed hydrocephalus varied significantly. Almost half of the respondents (45%) had full independence (> 81 points on the Barthel scale), while 27% were entirely dependent on their caregivers (total dependence). The children’s degree of independence varied significantly according to the etiology of hydrocephalus. The children with hemorrhagic hydrocephalus were the least independent. The results of research by Holwerda et al. confirmed this relationship, although that study used other tools to measure the functioning of children with hydrocephalus [2].

A more detailed analysis of the individual activities of everyday life, as assessed with the Barthel scale, indicated that approximately 30% of the patients had problems with urinating or defecating. The parents’ responses indicated that the children’s delays in learning self-care skills were most often the result of delayed psychomotor development, but systematic efforts produced the desired results. Such delays most frequently occurred in the children with posthemorrhagic hydrocephalus. Lack of urination or defecation control requires the constant use of hygienic diapers, which causes mental discomfort, especially among school-age children [3]. Problems with moving (on flat surfaces or on stairs) were present in 21% of the children, primarily those who used a wheelchair.

Approximately 13% of the children needed help from another person or equipment, e.g., crutches, to move (second-degree independence). The literature indicates that depending on the etiology of hydrocephalus, mobility disorders may affect between 12 and 38% of patients, including up to 93% [4, 5] of children with hydrocephalus related to MMC.

Among the most common additional disorders in the children that significantly reduced functional skills, the parents mentioned epilepsy, mobility disorders (e.g., MPD, myeloproliferative disorder), sensory deficits and cognitive function impairment. Epilepsy was present in approximately 30% of the children. Similar results were found by Vinchon, Rekate and Kulkarni and Persson, who reported that epilepsy was present in 6–34% of patients treated for hydrocephalus [6–8]. Mobility disorders due to paralysis of the upper or lower limbs, such as cerebral palsy, were present in 15% of the children with hydrocephalus. The literature indicates that cerebral palsy occurs in 3.8–27% of children with hydrocephalus [6, 8–10].

Visual disorders were reported for 13% of the examined children. In research presented by other authors, visual disorders were present in 12–83% of children with hydrocephalus. Children with epilepsy, MPD and posthemorrhagic hydrocephalus have a greater predisposition toward visual defects [10, 11]. Hearing problems were reported in 8% of the studied children with hydrocephalus. In research conducted by Panova, 24% of children treated for hydrocephalus were deaf, and hearing impairment or hearing loss was correlated with low quality of life, low birth weight and posthemorrhagic hydrocephalus [10, 12]. Cognitive impairment (delayed mental development, behavioral disturbance) was reported by 5% of the parents. The results of research conducted by Venkataraman and Mukundan showed that psychological development below the developmental norm affected 67% of children with hydrocephalus and 20% of children with MMC [4, 13]. The difference between their findings and the results of the present study may be related to the lack of a psychological diagnosis for a significant proportion of the children in the current study due to their age (3–6 years). All the disorders mentioned by the children’s parents are confirmed by the literature, along with the possibility of endocrine disorders [13, 14], depression [14] and chronic pain [6, 7]. One should take into account the fact that almost half of the respondents in the present study did not have any additional diseases.

The existence of numerous scientific works on the IQ of children treated for hydrocephalus allows an objective assessment of their intellectual development [15]. However, there is a deficit of information on the education of children in specific types of educational facilities in Poland, which makes direct comparisons difficult. The results of this research complement existing knowledge by presenting the surveyed parents’ responses regarding the education of children treated for hydrocephalus. Most of the children attended generally accessible educational facilities (60%). This result coincides with Casey’s research, which showed that 59% of children attended public schools and the rest (most often those with post-inflammatory and post-hemorrhage hydrocephalus) attended special schools [16, 17].

The literature indicates that children with hydrocephalus have a greater predisposition toward learning difficulties [16]. The results of research confirm this thesis. It is worth noting that among the studied children treated for hydrocephalus, 45% did not show any problems with learning. Concentration difficulties and graphomotor issues were the most common learning difficulties reported for the examined children and were a source of anxiety for their parents. Some of the children also had problems with science and reading. These difficulties were also noted by Kulkarni, who emphasized that difficulties with reading comprehension and mathematical skills might be related to slower processing of visuo-spatial information [18]. Research by Persson et al. indicated that 47% of children with hydrocephalus had learning problems [8]. Such information allows parents to their children's educational possibilities based on other parents’ experiences, taking into account both their child’s abilities and the ability of specific educational facilities to adapt his/her needs.

For children with hydrocephalus, rehabilitation is a very important part of the process of acquiring independence. A child’s central nervous system is the most plastic in the first months of his/her life, so it is important to start rehabilitation as soon as possible [19]. It is worth emphasizing that children with hydrocephalus can follow an entirely normal pattern of development, including full independence, attendance of public schools and a lack of cognitive deficits. However, some children treated for hydrocephalus develop many comorbidities that result in total or partial dependence and require rehabilitation and attendance of special schools, as well as the reorganization of family life.

## Conclusions

The lowest level of independence was found for children with hemorrhagic hydrocephalus who had undergone repeated operations. The child’s degree of independence and the etiology of hydrocephalus determined the type of school the child attended. The most common learning problems among the studied children were difficulties with concentration and learning to read and write. The results indicate that rehabilitation plays a key role in the life of children with hydrocephalus.
